# Impact of ABO Blood Group on Vascular Complications and on Clinical and Functional Outcome After Aneurysmal Subarachnoid Hemorrhage

**DOI:** 10.3390/neurolint18060115

**Published:** 2026-06-10

**Authors:** Vera Marschal, Andreas Ziebart, Maryam Abdoullahi, Daniel Werkmann, Ralph König, Thomas Kapapa, Benjamin Mayer, Johannes Rosskopf, Lennart Marschal, Christian Rainer Wirtz, Andrej Pala, Gregor Durner

**Affiliations:** 1 Department of Neurosurgery, University Hospital Ulm, Albert-Einstein-Allee 23, 89081 Ulm, Germany; andreas.ziebart@uniklinik-ulm.de (A.Z.); thomas.kapapa@uni-ulm.de (T.K.); rainer.wirtz@bkh-guenzburg.de (C.R.W.); 2 Department of Neurosurgery, District Hospital Günzburg, 89312 Günzburg, Germany; maryam.abdoullahi@bkh-guenzburg.de (M.A.); daniel.werkmann@bkh-guenzburg.de (D.W.); ralph.koenig@uni-ulm.de (R.K.); andrej.pala@bkh-guenzburg.de (A.P.); gregor.durner@bkh-guenzburg.de (G.D.); 3 Institute of Epidemiology and Medical Biometry, University of Ulm, 89081 Ulm, Germany; benjamin.mayer@uni-ulm.de; 4 Department of Neuroradiology, District Hospital Günzburg, 89312 Günzburg, Germany; johannes.rosskopf@bkh-guenzburg.de; 5 Department of Internal Medicine, Klinikum Heidenheim gGmbH, 89522 Heidenheim, Germany; lennart.marschal@kliniken-heidenheim.de

**Keywords:** aneurysmal subarachnoid hemorrhage, ABO blood group, cerebral vasospasm, delayed cerebral ischemia, cognitive function

## Abstract

**Objective**: To evaluate whether ABO blood group is associated with venous thromboembolic events (VTEs), cerebral severe vasospasm (CSV), delayed cerebral ischemia (DCI), and clinical or cognitive outcomes after aneurysmal subarachnoid hemorrhage (aSAH). **Materials and Methods**: A retrospective observational two-center cohort study of collected registry data, including 169 patients treated between September 2021 and November 2025. Outcomes were compared across ABO subtypes using univariate testing and multivariable logistic regression. **Results**: No ABO subtype was independently associated with VTE (7.7%), CSV/DCI (21.9%), intracranial hemorrhage, or in-hospital mortality (all *p* > 0.05). Higher age (OR 1.08, 95% CI 1.031–1.144, *p* = 0.003) was independently associated with increased in-hospital mortality, whereas single peri-interventional antiplatelet therapy (PIAT) (OR 0.076, 95% CI 0.004–0.506, *p* = 0.029) was associated with lower in-hospital mortality. ABO blood group was not associated with functional outcome (mRS) or cognitive performance (MoCA) in this cohort. **Conclusions**: In this two-center retrospective cohort, no independent association between ABO blood group and early cerebrovascular complications, functional outcome, or cognitive outcome after aSAH was detected. These findings suggest that short-term prognosis may be more strongly influenced by established patient- and treatment-related factors, particularly age and single PIAT. Further studies with larger cohorts are warranted to clarify the potential effect of ABO blood group on outcomes after aSAH.

## 1. Introduction

Spontaneous subarachnoid hemorrhage (SAH), most commonly caused by the rupture of an intracranial aneurysm, remains one of the most devastating cerebrovascular emergencies. With a sudden onset and a high risk of mortality and long-term disability, SAH affects approximately nine out of every 100,000 individuals per year, typically presenting at a mean age of 55 years [1,2,3]. While early management aims to prevent rebleeding and secure the aneurysm, secondary complications, such as cerebral severe vasospasm (CSV), delayed cerebral ischemia (DCI), and thromboembolic events (TEs), play a major role in long-term outcomes.

In recent years, the ABO blood group system has gained attention as a potential modifier of vascular and thrombotic risk in various clinical contexts. In the setting of SAH, several studies have investigated associations between ABO blood type and the development of DCI and vasospasm, though results remain inconsistent and sometimes contradictory [4,5,6,7,8]. The pathophysiological mechanisms underpinning these complications, such as microvascular dysfunction, endothelial injury, and inflammation, could plausibly be influenced by ABO-related variations in coagulation and vascular response.

Beyond cerebral complications, TEs, such as deep vein thrombosis (DVT) and pulmonary embolism (PE), also represent significant sources of morbidity and mortality in patients with SAH. Despite the well-established link between ABO blood type and thromboembolic risk in broader medical populations, this association remains largely underexplored in the context of aneurysmal SAH (aSAH). Individuals with non-O blood types have consistently been shown to have a higher risk of thromboembolic events, including venous thromboembolism (VTE) and post-surgical thrombosis due to higher levels of von Willebrand factor (vWF) and factor VIII (FVIII) [9,10].

Conversely, patients with blood group O generally exhibit lower levels of circulating vWF and FVIII, placing them at increased risk of bleeding in certain clinical scenarios, such as gastrointestinal bleeding or postpartum hemorrhage [11,12,13]. These differences are thought to reflect the expression of ABO antigens not only on red blood cells, but also on vascular endothelial cells and platelets, where they can influence hemostasis and endothelial function [14,15].

In the cerebral vasculature, these antigen-related differences might also modulate the risk of vasospasm and DCI, potentially through effects on microcirculatory perfusion, endothelial activation, or prothrombotic states [4,7]. However, high-quality evidence supporting such links remains limited, and available studies often lack adequate power or control for confounding variables.

Given the potential influence of ABO blood group on both thromboembolic risk and cerebrovascular complications following aSAH, a systematic investigation is warranted to clarify these associations. To address this, we conducted a retrospective observational study examining patients treated for spontaneous aSAH at two tertiary care centers. The study was designed to evaluate the relationship between ABO blood type and the occurrence of VTE and CSV/DCI, as well as to assess clinical, functional, and cognitive outcomes.

## 2. Materials and Methods

### 2.1. Study Design and Ethics Approval

This retrospective observational study was approved by the local ethics committee (Approval No. 280/21) and conducted in accordance with the Declaration of Helsinki. Written informed consent for the use of anonymized clinical data was obtained from all patients or their legal representatives.

### 2.2. Patient Population

**Figure 1:**
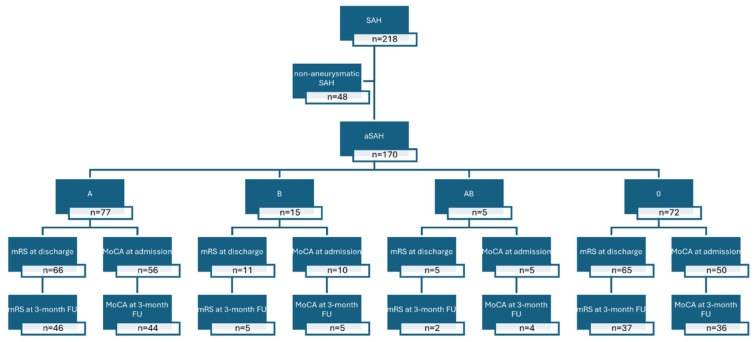
Study flowchart and availability of outcome assessments. Abbreviations: SAH = subarachnoid hemorrhage; aSAH = aneurysmal subarachnoid hemorrhage; mRS = modified Rankin Scale; MoCA = Montreal Cognitive Assessment; FU = follow-up.

A total of 173 patients diagnosed with spontaneous aSAH and treated at the District Hospital Günzburg or the University Hospital Ulm between September 2021 and November 2025 were included. Patients with non-aneurysmal, prepontine or traumatic SAH were excluded. A total of 4 patients from the 173 patients were excluded because of missing ABO blood group data (*n* = 169). The patients were categorized by ABO type. A cohort overview is shown in Figure 1. No formal sample size calculation was performed due to the exploratory retrospective design.

### 2.3. Baseline Characteristics Assessment

Baseline data included age, sex, rhesus factor, use of antiplatelet or anticoagulant agents at admission, smoking status, and comorbidities, such as arterial hypertension (AHT), diabetes mellitus (DM), previous TE, coronary artery disease (CAD), and malignancy. Other pre-existing conditions, as well as the absence of comorbidities, were recorded. Thrombophilic diatheses were documented in a single patient, who, however, experienced neither recurrent bleeding during the hospital stay nor a CV/DCI nor any VTE. Consequently, this pre-existing condition was not included in the analysis.

Neurological status on admission was assessed using the World Federation of Neurosurgical Societies (WFNSs) scale and the Hunt and Hess (HH) grading system [16,17]. SAH severity was categorized as mild (WFNS/HH grades I–III) or severe (grades IV–V). The extent of bleeding was assessed using the Fisher score (mild: grades 0–II; severe: grades III–IV) on initial imaging.

### 2.4. Diagnostic Imaging and Aneurysm Assessment

Diagnosis of SAH was confirmed via cranial computed tomography (CT), cranial magnetic resonance imaging (MRI), or lumbar puncture. The bleeding source and the presence of an aneurysm were determined using CT angiography (CTA) or digital subtraction angiography (DSA). All imaging was independently reviewed by at least one board-certified neuroradiologist and one experienced neurosurgeon.

The aneurysm identified as the source of the hemorrhage was classified according to its location within the anterior or posterior cerebral circulation. The anterior circulation included aneurysms of the anterior communicating artery, anterior cerebral artery, middle cerebral artery, internal carotid artery, anterior choroidal artery, and their distal branches and vascular territories. The posterior circulation included aneurysms of the posterior cerebral artery, posterior communicating artery, basilar artery, vertebral artery, posterior inferior cerebellar artery, superior cerebellar artery, labyrinthine artery, anterior inferior cerebellar artery, pontine arteries, and their respective vascular territories. Furthermore, the maximum aneurysm diameter was determined from imaging studies and recorded in millimeters (mm).

### 2.5. Aneurysm Treatment and Periprocedural Antithrombotic Therapy

The treatment modality, surgical or endovascular, was selected by a multidisciplinary neurovascular team, comprising at least one experienced neuroradiologist and one experienced vascular neurosurgeon. Post-procedural care was provided in the intensive care unit, with ongoing guidance and supervision from the neurovascular team throughout the patient’s hospitalization.

Microsurgical clipping was performed according to standard neurosurgical protocols.

Endovascular treatment included coiling, intrasaccular devices (e.g., Woven EndoBridge device (WEB) (MicroVention, Aliso Viejo, CA, USA), Contour Neurovascular System (Stryker, Portage, MI, USA), stents, and flow diverters. In all endovascular procedures, intra-arterial heparin (3000–5000 IU, weight-adjusted) was administered.

For some interventional procedures, patients received peri-interventional antiplatelet therapy (PIAT): Patients treated with intrasaccular devices alone (e.g., WEB) received oral acetylsalicylic acid monotherapy (100 mg daily) for six weeks [18,19]. In procedures involving stents or flow diverters, patients received dual antiplatelet therapy, consisting of a tirofiban infusion followed by prasugrel and acetylsalicylic acid. To prevent thromboembolic complications such as deep vein thrombosis, pulmonary embolism, or in-stent thrombosis, all hospitalized patients received standard prophylactic low-molecular-weight heparin within the first 24 h of admission, in accordance with institutional protocols. No cases of in-stent thrombosis were observed in this cohort.

### 2.6. Outcome Measures

#### 2.6.1. Venous Thromboembolism

VTE was defined as a new, radiologically confirmed diagnosis of PE, DVT, or cerebral venous thrombosis, occurring at any point during the neurosurgical inpatient stay. Imaging records for all patients were reviewed to identify investigations confirming any of these conditions. Only VTE events confirmed by radiological imaging were included in the analysis. Patients without radiological evidence of VTE were classified as not having experienced a VTE; however, imaging was performed based on clinical indication rather than as a routine screening procedure.

#### 2.6.2. Cerebral Severe Vasospasm and Delayed Cerebral Ischemia

CSV was defined as angiographic vessel narrowing on DSA or CTA, assessed independently by a board-certified neuroradiologist and a neurosurgeon. DCI was defined according to established criteria [20] as a new focal neurological deficit and/or a decrease in the level of consciousness not attributable to other causes, or as a DCI resulting in a new infarction confirmed on imaging.

#### 2.6.3. In-Hospital Mortality and Intracranial Hemorrhage

All deaths during hospitalization and newly diagnosed intracranial hemorrhages (excluding puncture channel-related hemorrhages secondary to external ventricular drainage) were systematically documented.

#### 2.6.4. Functional and Cognitive Outcome Assessment

Functional outcome was assessed using the modified Rankin Scale (mRS) at discharge and at the 3-month follow-up (FU) [21]. mRS 0–2 was classified as good (independent or partially dependent), and mRS 3–6 as poor (severely dependent to deceased). Cognitive performance was evaluated using the Montreal Cognitive Assessment (MoCA) during initial hospitalization (in non-intubated patients) and repeated at 3-month FU [22]. Patients scoring below 26 points were considered cognitively impaired. Patients unable to complete the MoCA due to a medical condition or death were assigned a score of zero. Missing MoCA and mRS data occurred due to loss to FU, as well as clinical and organizational factors inherent to routine care, including variability in physician staffing and documentation practices. In some cases, MoCA was not performed or recorded despite eligibility. Furthermore, a subset of patients was not testable due to a severe clinical condition.

### 2.7. Data Collection

Clinical, imaging, and laboratory data were extracted from electronic medical records and the institutional PACS, and compiled into a structured database (Microsoft Excel 2019, Redmond, WA, USA).

### 2.8. Statistical Analysis

Descriptive statistics summarized the baseline characteristics. Age was reported as mean ± standard deviation (SD), while aneurysm size was reported as median [interquartile range]. Group comparisons were performed using Student’s *t*-test, Kruskal–Wallis test or Mann–Whitney U test for continuous variables, and Fisher’s exact test or chi-square test for categorical variables. Categorical variables were presented as absolute numbers and percentages. Multivariate logistic regression was performed to identify independent associations between ABO blood group and the occurrence of VTE or CSV/DCI. Models were adjusted for potential confounders, including age, sex, initial neurological status (WFNS/HH grade), SAH severity (Fisher grade), and treatment modality (surgical vs. endovascular). Categorical variables were entered as dummy variables, with one category serving as the reference. Odds ratios (ORs) with 95% confidence intervals (CIs) were reported. A two-sided *p*-value ≤ 0.05 was considered statistically significant. Analyses were performed using R (Version 4.5.0) and Excel as complete-case analyses. Missing data were not imputed.

## 3. Results

### 3.1. Baseline Characteristics

Baseline characteristics stratified by ABO blood group are summarized in Table 1. No statistically significant differences were observed between blood groups with respect to demographic variables, clinical severity at admission, radiological hemorrhage burden, or treatment modality. Age, sex distribution, and rhesus factor were comparable across groups (all *p* > 0.05).

Initial neurological status assessed by WFNS and HH grading, as well as hemorrhage severity according to the Fisher score, did not differ significantly among patients with blood groups A, B, AB, or O. Likewise, the proportion of patients presenting with severe aSAH (WFNS/HH IV–V) was similar across all groups.

The treatment strategy (microsurgical clipping vs. endovascular intervention) and peri-interventional antithrombotic regimens, including mono- or dual-antiplatelet therapy, showed no significant association with ABO blood group. Regarding comorbidities, a non-significant trend toward a higher prevalence of CAD was observed in non-O blood groups (*p* = 0.055), while all other comorbid conditions were evenly distributed.

Medication at admission, including antiplatelet or anticoagulant therapy, did not differ significantly between ABO blood groups. Smoking status and prior VTE were also comparable.

### 3.2. Early In-Hospital Complications

Rates of early in-hospital complications, including CSV/DCI, VTE, new intracranial hemorrhage, and in-hospital mortality, were comparable across all ABO blood groups (Table 1).

Secondary univariate analyses comparing patients with and without CSV/DCI or VTE revealed no significant associations with ABO blood group or Rhesus factor (Table 2). There was no difference in aneurysm size between patients with and without VTE (Wilcoxon rank-sum test, *p* = 0.976). Similarly, aneurysm size did not differ between patients with and without mortality (*p* = 0.205), CSV/DCI (*p* = 0.107), or new intracranial hemorrhage (*p* = 0.153).

**Figure 2:**
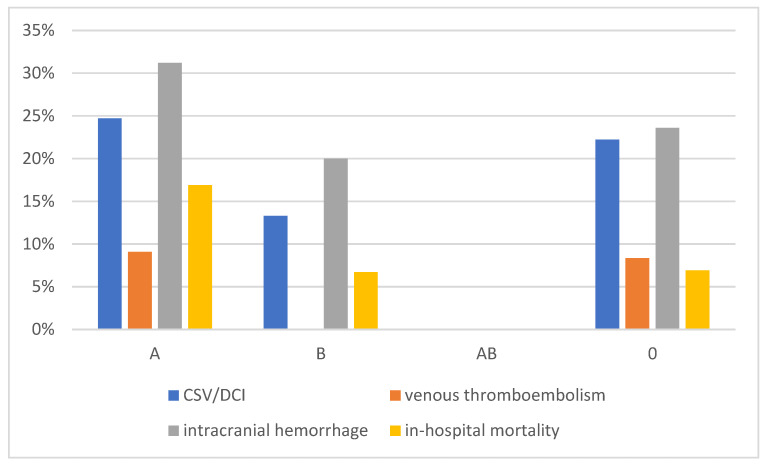
Complications per ABO blood group.

Figure 2 illustrates the distribution of major complications (cerebral severe vasospasm/delayed cerebral ischemia, venous thromboembolism, intracranial hemorrhage, and in-hospital mortality) across ABO blood groups. Due to the very small number of patients in the AB group (*n* = 5), these values should be interpreted with caution and are presented for descriptive purposes only. No inferential comparisons between groups are intended based on this visualization.

The AB blood group comprised only five patients, limiting the statistical interpretability of subgroup-specific complication rates. Therefore, comparisons involving this group should be interpreted cautiously and are primarily descriptive in nature.

### 3.3. Multivariable Analysis of Clinical Outcomes

Multivariable logistic regression models were performed in the complete cohort of 169 patients. The number of events was 13 for venous thromboembolism, 37 for vasospasm/DCI, 19 for in-hospital mortality, and 44 for hemorrhagic complications. After adjustment for clinically relevant SAH-related covariates, including age, sex, hemorrhage severity, clinical grade, treatment modality, and antiplatelet therapy, ABO blood group was not independently associated with any evaluated clinical outcome.

Specifically, no significant associations were found between ABO subtype and the occurrence of new intracranial hemorrhage (Table 3) or CSV/DCI (Table 4). None of the included covariates demonstrated a consistent independent effect on these endpoints after adjustment.

In contrast, multivariable analysis of in-hospital mortality identified increasing age as an independent predictor of death (OR 1.08, 95% CI 1.031–1.144, *p* = 0.003). Single antiplatelet therapy was associated with reduced mortality risk (OR 0.076, 95% CI 0.004–0.506, *p* = 0.029). ABO blood group showed no statistically significant association with mortality, although blood group A demonstrated a non-significant numerical trend toward increased risk (Table 5).

Regarding venous thromboembolism, the ABO blood group was again not independently associated with VTE occurrence. Also, all other covariates, including age, sex, treatment modality, hemorrhage severity, and antiplatelet strategy, were not significantly associated with VTE (Table 6).

To assess the robustness of the findings and address concerns regarding model overfitting, additional reduced multivariable logistic regression models, including only clinically relevant covariates, were performed for intracranial hemorrhage, VTE, vasospasm/DCI, and in-hospital mortality. Results remained consistent with the primary analyses, with no significant association between ABO blood group and any of the investigated outcomes (Supplementary Table S1).

Additional O versus non-O sensitivity analyses yielded comparable results (Supplementary Table S2) for venous thromboembolism (OR 1.06, 95% CI 0.36–3.34, *p* = 0.915), vasospasm/DCI (OR 1.06, 95% CI 0.50–2.23, *p* = 0.873), in-hospital mortality (OR 2.05, 95% CI 0.67–7.20, *p* = 0.229), or bleeding complications (OR 0.81, 95% CI 0.39–1.66, *p* = 0.570).

### 3.4. Functional and Cognitive Outcome

Cognitive outcomes assessed by the MoCA were incomplete at both assessment time points. At admission, MoCA data were available for 85 patients. Among the remaining patients, 36 were intubated and, therefore, not testable, while 48 had no documented assessment. At the 3-month follow-up, MoCA data were available for 86 patients. Missing assessments were attributable to death (*n* = 16), unavailable testing despite follow-up (*n* = 10), or loss to follow-up (*n* = 57). Analyses showed no significant differences between ABO blood groups at admission or at the 3-month follow-up (Table 7). The proportion of patients with cognitive impairment (MoCA ≤ 25) was comparable across blood group subtypes at both time points, with no relevant deviations in odds ratios relative to the reference group. A sensitivity analysis restricted to patients with available 3-month MoCA assessments (*n* = 86) yielded results consistent with the primary analysis. No association between ABO blood group and cognitive outcome was observed in either univariate (*p* = 0.761) or multivariable analyses (Supplementary Table S3).

Similarly, the functional outcome measured by the mRS did not differ significantly between ABO blood groups at discharge or at the 3-month follow-up (Table 8). The rates of favorable outcome (mRS 0–2) were comparable across all groups, and no statistically significant associations were observed in univariate analyses.

### 3.5. Summary of Outcome Analyses

Across all evaluated endpoints, including early vascular complications, in-hospital mortality, functional outcome, and cognitive performance, ABO blood group was not associated with adverse clinical or neurological outcomes in either univariate or multivariable analyses. Outcome variability in this cohort was primarily driven by established patient- and treatment-related factors, particularly age and mono PIAT, rather than ABO phenotype.

## 4. Discussion

Our findings are consistent with previous studies reporting no independent association between ABO blood group and outcomes after aSAH [5,8,23]. Specifically, no independent associations were observed between ABO subtype and the occurrence of VTE, CSV, DCI, intracranial hemorrhage, or in-hospital mortality, despite rigorous multivariable adjustment. As a sensitivity analysis, patients were additionally grouped into blood group O and non-O categories, reflecting the established biological differences in vWF and FVIII levels. This analysis yielded results consistent with the primary ABO subgroup analyses and did not reveal significant associations with TE, SV/DCI, bleeding complications, or in-hospital mortality. Although numerically higher mortality rates were observed in non-O blood groups, the association did not remain statistically significant after adjustment, and confidence intervals remained wide, indicating limited precision.

Instead, traditional clinical and treatment-related variables, most notably age and single PIAT for in-hospital mortality, emerged as more influential determinants. Age was identified as an independent predictor of in-hospital mortality in our cohort, consistent with prior studies demonstrating that older patients with aSAH have a higher risk of early death [2,20]. Interestingly, the single PIAT was independently associated with lower in-hospital mortality in our cohort. However, this finding should be interpreted with caution, given the observational and retrospective nature of the study. The observed association does not establish causality and may reflect residual confounding, treatment-selection effects, or differences in baseline clinical characteristics between patients who did and did not receive single PIAT. For example, antiplatelet therapy may have been administered preferentially in specific aneurysm subtypes, treatment settings, or patient groups with differing prognostic profiles. Survival bias cannot be excluded either, as patients must survive long enough to receive and maintain treatment. Nevertheless, our findings are consistent with recent meta-analyses reporting associations between post-ictal antiplatelet therapy and lower rates of delayed cerebral ischemia, symptomatic vasospasm, and in-hospital mortality following aSAH [24,25,26]. Therefore, the present observation should be regarded as hypothesis-generating and warrants further investigation in prospective studies specifically designed to evaluate the causal impact of antiplatelet strategies after aSAH.

Despite these findings, no significant differences in functional outcomes (mRS at discharge or 3-month FU) or cognitive performance (MoCA) were observed across ABO blood groups, suggesting that ABO-related hemostatic variations do not translate into measurable neurological or cognitive deficits in the early post-aSAH period. While most previous research on aSAH has focused on mortality or vasospasm, data on cognitive sequelae remain limited. Our results align with emerging evidence that ABO-driven hemostatic differences may influence acute thrombotic or bleeding risk, yet appear insufficient to affect longer-term neurological recovery or cognitive trajectories [22,23,27]. Future studies incorporating structured cognitive assessments may help identify more subtle associations not captured by traditional outcome measures.

These results are consistent with one large published study in this field by Wang and colleagues, which analyzed 663 aSAH patients and found no association between ABO blood type and DCI, mortality, Glasgow Outcome Score, ICU length of stay, or duration of hospital stay. In that study, baseline severity markers, such as Glasgow Coma Scale, WFNS, and Fisher grade, were also evenly distributed across blood groups, and multivariate analyses confirmed the absence of ABO effects on outcomes [8].

Earlier work has similarly reported null findings. A retrospective study of 470 aSAH patients found no significant relationship between ABO blood group and onset of vasospasm, SAH-associated intracerebral hemorrhage, DCI or admission severity scores (e.g., WFNS, Fisher) [5]. In contrast, smaller studies with limited statistical adjustment have reported conflicting results, for example, associations of blood group O with increased DCI [4,7] or blood group A with delayed ischemic neurological deficit [6]. However, these studies often lacked robust multivariable control for confounders, including age, aneurysm severity, and treatment variables, which limits interpretability and may explain discrepancies in effect estimates.

Pathophysiological rationale for a putative ABO effect lies predominantly in the hemostatic and vascular roles of ABO antigens. Non-O blood types are genetically associated with higher plasma levels of vWF and FVIII, largely mediated by differences in glycosylation of vWF molecules, which influence their clearance and functional activity [12,14]. Elevated vWF and FVIII are well documented to increase susceptibility to VTE in general populations, possibly through enhanced platelet adhesion and aggregation at sites of endothelial injury [28]. These mechanistic observations have been supported by large epidemiological investigations, demonstrating quantitative differences in vWF and FVIII distribution by ABO genotype in thousands of subjects [29,30,31], although such differences have not translated into consistent clinical effects within aSAH cohorts.

Nevertheless, the biology underpinning DCI and vasospasm after subarachnoid hemorrhage is multifactorial and cannot be solely explained by baseline variations in procoagulant proteins. Cerebral ischemic processes after aSAH involve a complex interplay between endothelial dysfunction, inflammatory cascades, oxidative stress, impaired cerebral autoregulation, and microvascular thrombosis [20]. In addition to coagulation-related pathways, inflammatory mechanisms may contribute to the outcome after aSAH. Elevated C-reactive protein levels have previously been associated with delayed cerebral ischemia, unfavorable functional outcome, and increased mortality after aSAH [32,33]. Within this setting, potential ABO-related differences in hemostasis may be relatively small compared with the influence of established clinical factors and contemporary treatment strategies. Consistent with this interpretation, no independent association between ABO blood group and clinically relevant outcomes was detected in the present cohort.

Despite the growing literature, several limitations remain. Many existing studies, including ours, are observational and retrospective, and residual confounding cannot be entirely excluded. The generalizability of findings may be limited by population structure; for example, genetic linkage between ABO and other risk loci varies across ethnicities, potentially affecting both hemostatic traits and vascular disease susceptibility. Large prospective and multiethnic cohorts with standardized outcome definitions (e.g., DCI, confirmed by imaging and clinical criteria) and comprehensive hemostatic profiling are needed to clarify whether any modest ABO effects exist in specific subgroups.

In clinical practice, our results, in line with large contemporary data, suggest that the ABO blood group should not presently influence individualized risk stratification or therapeutic decision-making in aSAH. Established predictors, such as age, admission severity scores, and early complications, remain the most robust guides for prognosis. Although the ABO group remains a biologically intriguing marker of hemostatic variation, its role in the pathophysiology of aSAH complications appears limited in magnitude and clinical impact relative to other factors that drive injurious cerebral and systemic responses.

This study has several limitations that should be considered when interpreting the results. The sample size, particularly in blood groups B and AB, limits the feasibility of detailed subgroup analyses and reduces statistical power for detecting smaller effect sizes. The multivariable regression analyses were exploratory and included clinically relevant covariates selected based on established prognostic factors in aSAH. As this was a retrospective observational study, no formal sample size calculation was performed. Consequently, the study may have been underpowered to detect smaller effect sizes, particularly within the less frequent ABO blood group subgroups, particularly for analyses involving blood group AB and rare clinical outcomes. The retrospective study design is associated with an inherent risk of residual confounding despite statistical adjustment. In addition, the follow-up was limited to the early recovery phase at three months, precluding conclusions about long-term outcomes. An additional limitation relates to the assessment of VTE. Radiological screening for VTE was not performed systematically in all patients but was generally based on clinical suspicion. As a result, asymptomatic or clinically unrecognized VTE events may have remained undetected. Patients who did not undergo radiological evaluation were classified as not having VTE, which may have led to underestimation of the true VTE incidence and introduced detection bias. Consequently, the ability to identify potential associations between the ABO blood group and VTE risk may have been reduced. Finally, the lack of laboratory data on inflammatory biomarkers, vWF, FVIII, or other coagulation parameters limits mechanistic insights into the interplay between ABO blood group, inflammatory status, and hemostatic pathways. The very small number of patients with blood group AB (*n* = 5) further limits the power to detect subtype-specific associations. These factors may have reduced the sensitivity to identify subtle but clinically relevant associations.

In summary, no independent association between the ABO blood group and early vascular complications, mortality, functional outcome, or cognitive outcome was detected in this cohort of patients with aSAH. Instead, conventional prognostic determinants, most prominently patient age and single PIAT for in-hospital mortality, remained the primary drivers of early prognosis. Although ABO-related differences in hemostatic pathways provide a plausible biological rationale, these differences did not translate into clinically meaningful short-term outcome differences in the present cohort [12,14,28,29,30,31]. Future research should incorporate larger multicenter cohorts, standardized coagulation and inflammatory biomarker profiling, and extended longitudinal follow-up to determine whether ABO-related differences may emerge beyond the acute treatment phase. Prospective study designs with mechanistic endpoints may be necessary to clarify whether phenotype-specific hemostatic or vascular properties become clinically relevant over time.

## Figures and Tables

**Table 1:** Patient cohort baseline characteristics by ABO blood group in univariate analysis.

Characteristics	A	B	AB	O	*p*-Value
**Number of patients**	77 (45.6%)	15 (8.9%)	5 (3.0%)	72 (42.6%)	
**Distribution in local blood bank**	43%	11%	5%	41%	
**Rhesus factor positive**	81.8% (63)	93.3% (14)	80.0% (4)	81.9% (59)	0.757
**Sex**					
Male	27.3% (21)	33.3% (5)	20.0% (1)	26.4% (19)	0.950
Female	72.7% (56)	66.7% (10)	80.0% (4)	73.6% (53)	
**Mean age (SD, min-max)**	58.1 (13.1 26–85)	58.9 (12.7 36–83)	54.8 (15.6 36–79)	56.5 (12.6 30–84)	0.730
**WFNS grade**					
I–III	51.9% (40)	53.3% (8)	100% (5)	58.3% (42)	0.203
IV–V	48.1% (37)	46.7% (7)	0% (0)	41.7% (30)	
**Hunt &Hess grade**					
I–III	64.9% (50)	60.0% (9)	100% (5)	70.8% (51)	0.372
IV–V	35.1% (27)	40.0% (6)	0% (0)	29.2% (21)	
**Fisher Score**					
0–II	13.0% (10)	20.0% (3)	40.0% (2)	13.9% (10)	0.310
III–IV	87% (67)	80.0% (12)	60.0% (3)	86.1% (62)	
**Aneurysm location**					
Anterior	66.2% (51)	66.2% (51)	66.2% (51)	66.2% (51)	0.480
Posterior	86.7% (13)	86.7% (13)	86.7% (13)	86.7% (13)	
**Median aneurysm diameter (mm) [IQR]**	6.0 [4.75–10]	6.0 [5.0–8.0]	8.0 [8.0–10.0]	6.5 [4.75–10.0]	0.310
**Treatment of aneurysmal SAH**					
Clipping	26.0% (20)	26.7% (4)	20.0% (1)	34.7% (25)	0.681
Endovascular treatment	74.0% (57)	73.3% (11)	80.0% (4)	65.3% (47)	
**PIAT**					
Single PIAT	26.0% (20)	20.0% (3)	40.0% (2)	19.4% (14)	0.396
Dual PIAT	22.1% (17)	26.7% (4)	40.0% (2)	16.7% (12)	
**Smoking status**					
Smoker	71.4% (22)	33.3% (5)	0% (0)	65.3% (25)	0.463
Non smoker	28.6% (55)	66.7% (10)	100% (5)	34.7% (47)	
**Health condition**					
CAD	3.9% (3)	13.3% (2)	20.0% (1)	1.4% (1)	0.055
AHT	44.2% (34)	33.3% (5)	40.0% (2)	47.2% (34)	0.821
DM	2.6% (2)	13.3% (2)	0% (0)	2.8% (2)	0.215
Malignoma	3.9% (3)	13.3% (2)	20.0% (1)	4.2% (3)	0.145
Prior VTE	9.1% (7)	6.7% (1)	20.0% (1)	8.3% (6)	0.673
Other	29.9% (23)	26.7% (4)	40.0% (2)	33.3% (24)	0.881
None known	27.3% (21)	13.3% (2)	40.0% (2)	19.4% (14)	0.379
**Antiplatelet therapy at admission**	10.4% (8)	26.7% (4)	0% (0)	13.9% (10)	0.339
**Anticoagulation at admission**	3.9% (3)	0% (0)	20.0% (1)	0% (0)	0.059
**Complications**					
VTE	7.8% (6)	0% (0)	0% (0)	9.7% (7)	0.796
CSV/DCI	24.7% (19)	13.3% (2)	0% (0)	22.2% (16)	0.667
New intracranial bleeding	31.2% (24)	20.0% (3)	0% (0)	23.6% (17)	0.444
In-hospital mortality	16.9% (13)	6.7% (1)	0% (0)	6.9% (5)	0.262

Abbreviations: AHT = arterial hypertension; DM = diabetes mellitus; CAD = coronary artery disease; CSV = cerebral severe vasospasm; DCI = delayed cerebral ischemia; IQR = interquartile range; PIAT = peri-interventional antiplatelet therapy; SAH = subarachnoid hemorrhage; VTE = venous thromboembolism; WFNS = World Federation of Neurosurgical Societies scale.

**Table 2:** Univariate comparison of baseline characteristics in patients with and without cerebral severe vasospasm (CSV)/delayed cerebral ischemia (DCI) or venous thromboembolism (VTE).

Characteristics	CSV/DCI	No CSV/DCI	*p*-Value	VTE	No VTE	*p*-Value
**Number of patients**	37 (21.9%)	132 (78.1%)		13 (7.7%)	156 (92.3%)	
**Blood group**						
A	51.4% (19)	43.9% (58)	0.459	53.8% (7)	44.9% (70)	0.573
B	5.4% (2)	9.8% (13)	0.527	0% (0)	9.6% (15)	0.609
AB	0% (0)	3.8% (5)	0.587	0% (0)	3.2% (5)	>0.99
O	43.2% (16)	23.4% (56)	>0.99	46.2% (6)	42.3% (66)	0.780
**Rhesus factor**						
positive	81.1% (30)	83.3% (110)	0.806	76.9% (10)	83.3% (130)	0.470
negative	18.9% (7)	16.7% (22)		23.1% (3)	16.7% (26)	
**Sex**						
Male	27.0% (10)	27.2% (36)	>0.99	53.8% (7)	25.0% (39)	**0.046**
Female	73.0% (27)	72.7% (96)		46.2% (6)	75.0% (117)	
**Mean age (SD, min-max)**	59.7 (13.0, 35–84)	56.7 (12.8, 26–85)	0.223	55.9 (9.4, 38–73)	57.5 (13.1, 26–85)	0.560
**WFNS grade**						
I–III	51.4% (19)	57.6% (76)	0.575	69.2% (9)	55.1% (86)	0.393
IV–V	48.6% (18)	42.4% (56)		30.8% (4)	44.9% (70)	
**Hunt & Hess grade**						
I–III	67.6% (25)	68.2% (90)	>0.99	69.2% (9)	68.0% (106)	>0.99
IV–V	32.4% (12)	31.8% (42)		30.8% (4)	32.1% (50)	
**Fisher Score**						
0–II	13.5% (5)	15.2% (20)	>0.99	15.4% (2)	14.7% (23)	>0.99
III–IV	86.5% (32)	84.8% (112)		84.6% (11)	85.3% (133)	
**Treatment of aneurysmal SAH**						
Clipping	18.9% (7)	32.6% (43)	0.153	30.8% (4)	29.5% (46)	>0.99
Endovascular treatment	81.1% (30)	67.4% (89)		69.2% (9)	70.5% (110)	
**PIAT**						
Single PIAT	32.4% (12)	20.5% (27)	0.175	15.4% (2)	23.7% (37)	0.856
Dual PIAT	24.3% (9)	19.7% (26)		23.1% (3)	20.1% (32)	
**Smoking status**						
Smoker	18.9% (7)	34.1% (45)	0.106	30.8% (4)	30.8% (48)	>0.99
Non smoker	81.1% (30)	65.9% (87)		69.2% (9)	69.2% (108)	
**Health condition**						
CAD	8.1% (3)	3.0% (4)	0.178	0% (0)	4.5% (7)	>0.99
AHT	45.9% (17)	43.9% (58)	0.853	53.8% (7)	43.6% (68)	0.566
DM	2.7% (1)	3.8% (5)	>0.99	0% (0)	3.8% (6)	>0.99
Malignoma	2.7% (1)	6.1% (8)	0.685	7.7% (1)	5.1% (8)	0.522
Prior VTE	8.1% (3)	9.1% (12)	>0.99	7.7% (1)	9.0% (14)	>0.99
Other	29.7% (11)	31.8% (42)	>0.99	23.1% (3)	32.1% (50)	0.757
None known	27.0% (10)	22.0% (29)	0.515	23.1% (3)	23.1% (36)	>0.99
**Antiplatelet therapy at admission**	18.9% (7)	11.4% (15)	0.269	7.7% (1)	13.5% (21)	>0.99
**Anticoagulation at admission**	0% (0)	3.0% (4)	0.577	0% (0)	2.6% (4)	>0.99

Abbreviations: AHT = arterial hypertension; DM = diabetes mellitus; CAD = coronary artery disease; PIAT = peri-interventional antiplatelet therapy; SAH = subarachnoid hemorrhage; VTE = venous thromboembolism; WFNS = World Federation of Neurosurgical Societies scale. Statistically significant values (*p* < 0.05) are shown in **bold**.

**Table 3:** Multivariable logistic regression for intracranial hemorrhage: Association of ABO blood group and clinical covariates.

	Odds Ratio	95% CI	*p*-Value
A vs. O	0.715	0.327–1.542	0.395
B vs. O	1.283	0.332–6.413	0.734
AB vs. O	n.e.	n.e.	0.988
Single PIAT	0.611	0.211–1.737	0.355
Dual PIAT	0.934	0.300–3.018	0.906
Fisher-Score	3.073	0.777–20.60	0.158
Age	0.980	0.950–1.009	0.181
Sex female	1.327	0.580–2.975	0.495
Endovascular treatment	1.681	0.639–4.565	0.297
Hunt & Hess grade IV–V	0.467	0.132–1.452	0.206
WFNS-Score IV–V	0.967	0.307–3.449	0.956

Abbreviations: PIAT = peri-interventional antiplatelet therapy; WFNS = World Federation of Neurosurgical Societies scale; n.e. = not estimable due to sparse data and quasi-complete separation.

**Table 4:** Multivariable logistic regression for cerebral severe vasospasm (CSV) and delayed cerebral ischemia (DCI). ABO blood group and secondary predictors.

	Odds Ratio	95% CI	*p*-Value
A vs. O	1.007	0.456–2.223	0.985
B vs. O	2.171	0.499–15.332	0.353
AB vs. O	n.e.	n.e.	0.988
Single PIAT	0.507	0.178–1.404	0.194
Dual PIAT	0.609	0.207–1.788	0.362
Fisher-Score	1.066	0.351–3.700	0.913
Age	0.980	0.950–1.010	0.191
Sex female	0.940	0.382–2.188	0.888
Endovascular treatment	0.632	0.204–1.862	0.410
Hunt & Hess grade IV–V	1.497	0.437–4.843	0.505
WFNS-Score IV–V	0.628	0.206–2.070	0.423

**Table 5:** Multivariable predictors of in-hospital mortality: ABO blood group and independent clinical risk factors.

	Odds Ratio	95% CI	*p*-Value
A vs. 0	2.445	0.756–8.962	0.149
B vs. 0	0.432	0.019–3.756	0.500
AB vs. 0	n.e.	n.e.	0.994
Single PIAT	0.076	0.004–0.506	**0.029**
Dual PIAT	1.289	0.322–5.082	0.714
Fisher-Score	0.541	0.081–4.660	0.535
Age	1.081	1.031–1.144	**0.003**
Sex female	1.321	0.359–5.865	0.690
Endovascular treatment	2.945	0.697–14.442	0.156
Hunt & Hess grade IV–V	5.079	0.069–36.692	0.087
WFNS-Score IV–V	2.135	0.234–15.446	0.460

Statistically significant values (*p* < 0.05) are shown in **bold**.

**Table 6:** Multivariable analysis of venous thromboembolism (VTE): ABO blood group and secondary predictors.

	Odds Ratio	95% CI	*p*-Value
A vs. O	1.058	0.312–3.522	0.926
B vs. O	n.e.	n.e.	0.992
AB vs. O	n.e.	n.e.	0.995
Single PIAT	0.359	0.068-	0.185
Dual PIAT	1.020	0.154–8.337	0.983
Fisher-Score	2.403	0.372–47.629	0.435
Age	0.980	0.934–1.027	0.405
Sex female	0.682	0.139–2.554	0.596
Endovascular treatment	0.529	0.065–3.459	0.505
Hunt & Hess grade IV–V	0.497	0.024–3.564	0.544
WFNS-Score IV–V	1.904	0.284–38.153	0.571

**Table 7:** Univariate analysis of cognitive outcomes (MoCA) at admission and the 3-month follow-up across ABO blood groups.

Characteristics	A		B		AB		O		*p*-Value
MoCA after admission									
	*n* = 56	OR (CI)	*n* = 10	OR (CI)	*n* = 5	OR (CI)	*n* = 50	OR (CI)	
≤25 points	83.9% (47)	0.846 (0.327–2.185)	80.0% (8)	1.211 (0.238–6.156)	60.0% (3)	3.404 (0.532–21.766)	84.0% (42)	0.850 (0.323–2.233)	0.499
≥26 points	16.1% (9)		20.0% (2)		40.0% (2)		16.0% (8)		
MoCA after 3 months									
	*n* = 44	OR (CI)	*n* = 5	OR (CI)	*n* = 4	OR (CI)	*n* = 36	OR (CI)	
≤25 points	68.2% (30)	1.429 (0.600–3.412)	80.0% (4)	2.340 (0.250–21.882)	50.0% (2)	0.545 (0.073–4.070)	58.3% (21)	0.661 (0.275–1.591)	0.671
≥26 points	31.8% (14)		20.0% (1)		50.0% (2)		41.7% (15)		

Abbreviations: MoCA = Montreal Cognitive Assessment.

**Table 8:** Univariate analysis of functional outcomes (mRS) at discharge and the 3-month follow-up across ABO blood groups.

Characteristics	A		B		AB		O		*p*-Value
mRS at discharge									
	*n* = 66	OR (CI)	*n* = 11	OR (CI)	*n* = 5	OR (CI)	*n* = 65	OR (CI)	
0–2	43.9% (29)	0.803 (0.418–1.543)	36.4% (4)	0.624 (0.175–2.231)	100% (5)	n.e.	47.7% (31)	1.056 (0.550–2.027)	0.096
3–6	56.1% (37)		63.6% (7)		0% (0)		52.3% (34)		
mRS at 3-month follow-up									
	*n* = 46	OR (CI)	*n* = 5	OR (CI)	*n* = 2	OR (CI)	*n* = 37	OR (CI)	
0–2	52.2% (24)	0.755 (0.328–1.739)	60.0% (3)	1.213 (0.193–7.633)	100% (2)	n.e.	56.8% (21)	1.086 (0.466–2.531)	0.786
3–6	47.8% (22)		40.0% (2)		0% (0)		43.2% (16)		

Abbreviations: mRS = modified Rankin Scale; n.e. = not estimable due to sparse data and quasi-complete separation.

## Data Availability

The original contributions presented in this study are included in the article. Raw data are available upon reasonable request, subject to ethical and data protection regulations. Further inquiries can be directed to the corresponding author.

## Supplementary Materials

The following supporting information can be downloaded at: https://www.mdpi.com/article/10.3390/neurolint18060115/s1, Table S1: sensitivity reduced multivariable logistic regression models for intracranial hemorrhage, VTE, vasospasm/DCI, and in-hospital mortality; Table S2: O versus non-O sensitivity analyses for intracranial hemorrhage, VTE, vasospasm/DCI, and in-hospital mortality; Table S3: multivariable analysis of dichotomized MoCA (<26 versus ≥26) at the 3-month follow-up.

## Abbreviations

ABO	ABO blood group system
aSAH	aneurysmal subarachnoid hemorrhage
AHT	arterial hypertension
CAD	coronary artery disease
CI	confidence interval
CSV	cerebral severe vasospasm
CT	computed tomography
CTA	computed tomography angiography
DCI	delayed cerebral ischemia
DM	diabetes mellitus
DSA	digital subtraction angiography
DVT	deep vein thrombosis
FVIII	factor VIII
HH	Hunt and Hess scale
IQR	interquartile range
MRI	magnetic resonance imaging
mRS	modified Rankin Scale
MoCA	Montreal Cognitive Assessment
OR	Odds ratio
PE	pulmonary embolism
PIAT	peri-interventional antiplatelet therapy
SAH	subarachnoid hemorrhage
SD	standard deviation
TE	thromboembolic events
VTE	venous thromboembolism
vWF	von Willebrand factor
WEB	Woven EndoBridge device
WFNS	World Federation of Neurosurgical Societies scale
