# Supplementary material for: Impact of ABO Blood Group on Vascular Complications and on Clinical and Functional Outcome After Aneurysmal Subarachnoid Hemorrhage
Source: Neurol Int. 2026 Jun 10;18(6):115. doi: 10.3390/neurolint18060115 (PMC13306091; doi:10.3390/neurolint18060115)
Supplement: Supplementary file 1 [file neurolint-18-00115-s001.zip › neurolint-4301241-supplementary.pdf]

Supplementary Table S1: sensitivity reduced multivariable logistic regression models for intracranial hemorrhage, VTE, vasospasm/DCI, and in-hospital mortality

Outcome	events / total	comparison (vs. blood group O)	Covariates	OR (non-O vs O)	95% CI	p-value
intracranial hemorrhage	44/169	Blood group A	age, sex, PIAT	0.70	0.33 - 1.46	0.341
		Blood group B		1.25	0.34 - 6.03	0.751
		Blood group AB		not estimable*	-	0.988
vasospasm/DCI	37/169	Blood group A	age, Fisher grade	0.90	0.41 - 1.93	0.781
		Blood group B		1.94	0.47 - 13.33	0.416
		Blood group AB		not estimable*	-	0.989
in-hospital mortality	19/169	Blood group A	age, Hunt&Hess grade, PIAT	2.57	0.81 - 9.28	0.122
		Blood group B		0.56	0.03 - 4.54	0.633
		Blood group AB		not estimable*	-	0.994
venous thromboembolism	15/169	Blood group A	age, sex	1.03	0.32 - 3.40	0.954
		Blood group B		0.73	0.04 - 4.85	0.778
		Blood group AB		2.86	0.13 - 25.79	0.393

Abbreviations: CI = confidence interval; DCI = delayed cerebral ischemia; OR = Odds ratio

* Estimates for blood group AB were unstable because of the very small number of patients (n = 5), resulting in quasi-complete separation and unreliable confidence intervals.

Supplementary Table S2: O versus non-O sensitivity analyses for intracranial hemorrhage, VTE, vasospasm/DCI, and in-hospital mortality

Outcome	blood group O	blood group non-O	Covariates	OR (non-O vs O)	95% CI	p-value
intracranial hemorrhage	17/72 (23.6%)	27/97 (27.8%)	age, sex, PIAT	0.81	0.39 - 1.66	0.57
vasospasm/DCI	16/72 (22.2%)	21/97 (21.6%)	age, Fisher grade	1.06	0.50 - 2.23	0.873
in-hospital mortality	5/72 (6.9%)	14/97 (14.4%)	age, Hunt&Hess grade, PIAT	2.05	0.67-7.20	0.229
venous thromboembolism	6/72 (8.3%)	9/97 (9.3%)	age, sex	1.06	0.36 - 3.34	0.915

Supplementary table S3: multivariable analysis of dichotomized MoCA (<26 versus ≥26) at 3 months follow up

Variable	OR	95% CI	p-value
blood group A vs 0	1.26	0.46 - 3.45	0.648
blood group B vs 0	3.88	0.45 - 85.54	0.269
blood group AB vs 0	0.83	0.08 - 8.62	0.869
age	1.05	1.01 - 1.09	**0.020**
Hunt&Hess I-III	0.37	0.10 - 1.16	0.102
Fisher 0-2	0.45	0.11 - 1.71	0.249
